# Germline large genomic alterations on 7q in patients with multiple primary cancers

**DOI:** 10.1038/srep41677

**Published:** 2017-01-31

**Authors:** R. A. R. Villacis, T. R. Basso, L. M. Canto, A. F. Nóbrega, M. I. Achatz, S. R. Rogatto

**Affiliations:** 1 International Research Center (CIPE), A.C. Camargo Cancer Center, São Paulo, SP, Brazil; 2 Department of Genetics and Morphology, Institute of Biological Sciences, University of Brasília - UnB, Brasília, DF, Brazil; 3 Department of Oncogenetics, A.C. Camargo Cancer Center, São Paulo, Brazil; 4 Department of Clinical Genetics, Vejle Hospital, DK and University of Southern Denmark, Denmark; 5 Department of Urology, Faculty of Medicine, São Paulo State University (UNESP), Botucatu, São Paulo, Brazil

## Abstract

Patients with multiple primary cancers (MPCs) are suspected to have a hereditary cancer syndrome. However, only a small proportion may be explained by mutations in high-penetrance genes. We investigate two unrelated MPC patients that met Hereditary Breast and Ovaria Cancer criteria, both presenting triple negative breast tumors and no mutations in *BRCA1, BRCA2* and *TP53* genes. Germline rearrangements on chromosome 7q, involving over 40 Mb of the same region, were found in both patients: one with mosaic loss (80% of cells) and the other with cnLOH (copy-neutral loss of heterozygosity) secondary to maternal allele duplication. Five children tested had no alterations on 7q. The patients shared 330 genes in common on 7q22.1-q34, including several tumor suppressor genes (TSGs) previously related to breast cancer risk and imprinted genes. The analysis of the triple negative BC from one patient revealed a mosaic gain of 7q translated for over-expressed cancer-related genes. The involvement of TSGs and imprinted genes, mapped on 7q, has the potential of being associated to MPC risk, as well as cancer progression. To our knowledge, this is the first description of patients with MPCs that harbor constitutive large alterations on 7q.

The incidence of cancer is continuously increasing, as is the number of cancer survivors^1^,^2^. Cancer patients have a higher risk of developing new malignancies when compared to the general population^3^. Data from the Surveillance, Epidemiology and End Results program estimated that subsequent primary cancers represent approximately 18% of all cancers in the USA^4^. The development of multiple primary cancers (MPCs) has been reported as being associated to the treatment received for the first cancer (chemotherapy and radiotherapy), personal lifestyle and genetic predisposition^5^.

Individuals who developed cancer at younger age, presented multiple primary tumors or reported several relatives with neoplasms are suspected of having a hereditary cancer predisposition syndrome^6^. Breast cancer (BC) falls within the tumor spectrum of several hereditary diseases, including Hereditary Breast and Ovarian Cancer syndrome (HBOC) and Li-Fraumeni syndrome (LFS)^6^. However, only a small proportion of familial BC cases can be explained by mutations in high-penetrance genes, such as *BRCA1, BRCA2* and *TP53*^7^.

Part of the missing heritability in BC may be explained by copy number variations (CNVs), which are chromosomal regions altered by gains or losses, or by copy-neutral loss of heterozygosity (cnLOH), defined as homozygous regions appearing as a result of inheritance of a pair of alleles from a single parent^8^,^9^. The contribution of rare germline CNVs to BC predisposition has been demonstrated in *BRCA1*/*BRCA2* mutation-negative patients^10^,^11^,^12^. Moreover, an increased frequency of cnLOH in cases where no mutations are present in the mismatch repair genes suggests the involvement of unknown germline alterations in familial colorectal cancer risk^13^.

Deletions and cnLOH mapped on 7q have been widely described in both hematological malignancies; specifically myelodysplastic syndrome, acute myeloid leukemia (AML) and splenic marginal zone lymphoma^14^,^15^,^16^; and BC^17^,^18^. Furthermore, genomic deletions on chromosome 7q have also been associated with congenital defects, including developmental delay, learning difficulties, craniofacial dysmorphism and hypogenitalism^19^,^20^,^21^,^22^.

Herein, we report the molecular and clinical characterization of two unrelated MPC patients, both presenting triple negative BC, a positive family history of cancer, and without germline pathogenic mutations in *BRCA1, BRCA2* and *TP53* genes, showing large genomic rearrangements mapped on 7q.

## Results

### Patient 1 and relatives

**Figure 1:**
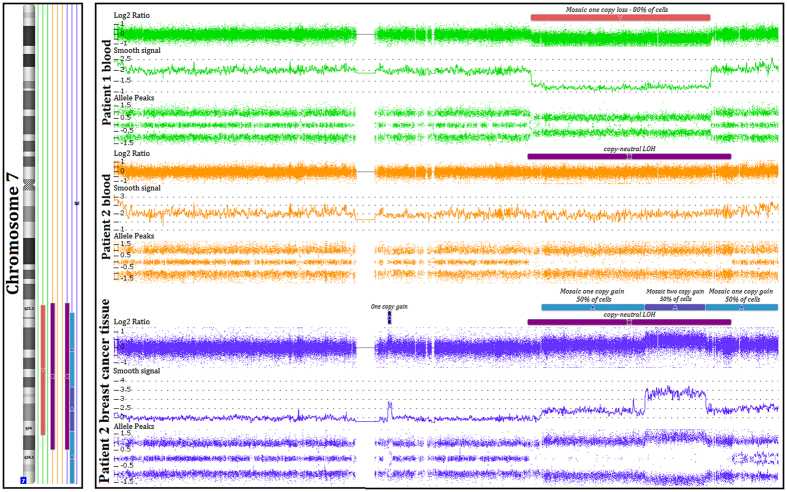
Schematic representation of the large alterations on chromosome 7q detected in Patient 1 (mosaic loss) and Patient 2 (cnLOH) using the Affymetrix CytoScan HD platform. All alterations were confirmed by non-polymorphic probes (Log2 Ratio and smooth signal) and SNP probes (allele peaks). In the breast cancer tissue of Patient 2, an additional gain at a different region of chromosome 7q was detected. Moreover, almost all of the cnLOH region presented a mosaic gain, particularly at the 7q32-q34 region.

The whole genomic analysis performed in the lymphocytic DNA from Patient 1 revealed a 43 Mb germline mosaic loss (80% of cells) of chromosome 7q22.1-q34 (Fig. 1) and a rare loss of 9q22.31 (Supplementary Table S1). Two children were evaluated for genomic alterations to assess the presence of 7q rearrangements. Her son inherited the rare deletion of 9q, while her daughter had only common CNVs. None of them presented any alteration of chromosome 7q (data not shown).

### Patient 2 and relatives

A large cnLOH (49 Mb) of 7q22.1-q36.1 was detected in the lymphocytic DNA of Patient 2 (Fig. 1). The region covered by the large mosaic loss of Patient 1 was entirely contained within the region encompassed by the cnLOH of Patient 2, both sharing 330 genes. An additional 76 genes were also mapped exclusively in the cnLOH region (Supplementary Table S1). Moreover, three other rare alterations were identified in Patient 2: loss of 8q11.21, cnLOH of 19p13.11-p13.2 and loss of Xq25 (Supplementary Table S1). Of them, losses of 8q11.21 and Xq25 were inherited from her mother. Among the three children tested for genomic alterations, the son A inherited the rare loss of 8q11.21 from Patient 2 (Supplementary Table S1). No alteration mapped in chromosome 7q was identified in all relatives tested.

The genotype analysis using the SNPs contained in the 7q cnLOH region of Patient 2 revealed its maternal origin. Virtually all homozygous nucleotides present in Patient 2, mapped to 7q22.1-q36.1, were identified in her mother, either homozygous or heterozygous (Supplementary Table S2).

The triple negative BC tissue of the Patient 2 presented a large number of alterations (100) in almost all chromosomes, including large CNVs and cnLOH regions in mosaicism (Supplementary Fig. S1 and Table S3). The germline 7q22.1-q36.1 cnLOH was maintained in the tumor tissue. However, a large percentage of this region also exhibited a gain in mosaicism in approximately 50% of cells (Fig. 1). Specifically, a region close to 15 Mb (7q32.1-q34) presented two copy gains in mosaicism.

The gene expression analysis performed in the BC tissue showed 96 over- and 52 down-regulated genes among the 406 genes contained in the 7q cnLOH region (Supplementary Table S4). At least 62 of the 148 differentially expressed genes were cancer-related according to the Candidate Cancer Gene Database (CCGD, http://ccgd-starrlab.oit.umn.edu/about.php)^23^, and the Network of Cancer Genes 5.0 (NCG, http://ncg.kcl.ac.uk/query.php)^24^. Among the 312 genes mapped in this region with mosaic gain, 81 were up-regulated and from these, 34 genes were cancer-related (Supplementary Table S4).

The kinship between the Patients 1 and 2 was verified using the Mendelian Error Check tool (ChAS software v3.1), which considers the genotypable SNPs (749,157 probes) found in the CytoScan HD platform. The analysis showed 5.8% of errors (of all SNPs) and role validity equals zero, thus confirming their unrelatedness.

## Discussion

The occurrence of MPCs in one individual may be due to environmental factors, aging, germline mutations or simply by chance^5^,^6^. We reported two unrelated patients with MPCs and family history of malignant neoplasms, being the triple negative BC the only tumor type common to both individuals. Of note, the emergence of three hematological malignancies (two lymphomas and leukemia) in Patient 1 occurred following treatment with radiotherapy and chemotherapy. The role of radio- and chemotherapy in subsequent malignant neoplasms is well recognized^5^. Myeloid neoplasms are among the most common cancers related to chemotherapy^5^.

Previous studies have reported a higher risk of lymphoma development following BC, as highlighted by Patient 1^25^, or the development of BC after melanoma, as observed in Patient 2^26^. However, the presence of multiple neoplasms, cancer at an early age (Patient 2) and a history of malignancy in several relatives strongly implies a hereditary factor as a cause of the cancer^6^. Indeed, both patients met the clinical criteria for HBOC^27^, having been tested for *BRCA1, BRCA2* and *TP53* mutations with negative results. A recent study evaluated 212 individuals with MPCs and identified a pathogenic variant in known cancer predisposition genes in less than 25% of the patients^28^.

In an effort to uncover the genetic basis of cancer in our patients, we performed molecular cytogenetic analyses. Large genomic rearrangements (over 40 Mb) on the long arm of chromosome 7 in both patients, starting at cytoband 7q22.1: a mosaic loss (80% of cells) in Patient 1 and a slightly larger DNA region with cnLOH in Patient 2. In a cohort of 100 health Brazilian individuals and 68 HBOC patients negative for mutations in *BRCA1* and *BRCA2*, Krepischi *et al*.^29^ found only a limited number of common CNVs (<500 Kb) mapped in 7q. To our knowledge, there are no previous reports in literature of patients with cancer and constitutive alterations as large as those described here.

Interestingly, large germline deletions on 7q ranging from 10 Mb to 30 Mb, most of them entirely contained within the deletion found in mosaicism in Patient 1, have been reported in individuals with a significant spectrum of clinical features^19^,^20^,^21^,^22^. Although Patient 1 presented haploinsufficiency of various genes related to congenital anomalies (e.g. learning difficulties, speech delay, craniofacial alterations, cardiac defects and growth retardation), it seems that the percentage of normal cells (20%) on the 7q region was sufficient to avoid the manifestation of any malformation. Moreover, tissues derived from embryonic germ layers other than the mesoderm (blood lymphocytes) may have different levels of mosaicism, which may help to explain the absence of malformations in this patient. Recently, we reported a case with multiple tumors and deletion of one X chromosome in mosaicism, which did not present the clinical phenotype of Turner syndrome^30^.

Gains and losses involving genomic regions containing oncogenes or tumor suppressor genes (TSGs) may lead to cancer development^8^. Rare copy number alterations have been implicated in BC susceptibility in HBOC patients negative for mutations in *BRCA1* and *BRCA2* genes^10^,^11^,^12^. In addition, cnLOH may also contribute to cancer risk since they not occur randomly across the genome, but are associated with mutations in key cancer-related genes^9^,^31^. Recently, an increased level of cnLOHs involving 7q was reported in familial colorectal patients negative for mismatch repair genes compared to sporadic cases^13^.

The triple negative BC sample from Patient 2 was evaluated by molecular cytogenetic and transcriptomic analysis to compare with the germline alterations and to verify if the presence of 7q alterations could modify the dosage of genes mapped in this region. An extremely large number of genomic alterations, mainly gains and losses in mosaicism were found, thereby highlighting intra-tumor heterogeneity, and clonal evolution. In addition to the cnLOH of 7q found in lymphocytic DNA, two new cnLOHs and 10 mosaic cnLOHs were observed in the tumor, therefore suggesting the importance of cnLOHs in BC progression, as described in colorectal cancer^32^. Interestingly, cnLOH regions were detected more frequently in triple negative BC samples than in estrogen/progesterone/HER2 positive cases^33^. Moreover, there are various common fragile sites in 7q, which are regions prone to genomic rearrangement and have been previously associated with cancer development^34^,^35^.

The BC sample transcriptome analysis revealed 148 genes differentially expressed mapped in the same region of 7q cnLOH, most of them over-expressed (96 genes) and cancer-related (62 genes). These results indicated that large genomic rearrangements on 7q alter the gene dosage and give additional support for the functional relevance of this alteration found in both patients of our study.

The detected 7q deletion and cnLOH shared a large number of genes (330), including putative TSGs previously associated with BC risk (e.g. *CAV1, MET* and *TES*)^18^,^36^. Down-regulation of *CAV1* gene was observed in the BC tissue of Patient 2. The presence of TSGs associated with hematological cancers, such as *DOCK4, LUC7L2* and *CUX1* were also observed^16^. Interestingly, Patient 1 developed two primary lymphomas and AML within 9 years. Recently, mutations in *CUX1* have been identified in 20 different carcinoma types, making this gene an important candidate for tumorigenesis^37^. In addition, the oncogene *BRAF*; previously reported as involved in melanoma, papillary thyroid carcinoma and colorectal cancer^38^; was included in the altered genomic region mapped to 7q and may be associated with the emergence of melanoma three years before the BC in Patient 2.

Besides its involvement in unmasking homozygous cancer-related mutations, cnLOHs may also disrupt genomic imprinting, an epigenetic process that leads to monoalleic expression depending on parental origin, resulting in two active or repressed alleles^9^,^39^. The deregulation of imprinted genes has been described in malignant neoplasms^40^. According to the Geneimprint database (http://www.geneimprint.com/site/genes-byspecies), five imprinted genes are mapped to 7q32: *COPG2IT1, MEST, CPA4, MESTIT1* and *KLF14*, of which, a loss of imprinting (biallelic expression) of *MEST* has been associated with BC development^40^,^41^,^42^. Interestingly, *MEST* was apparently silenced in the blood sample of Patient 2 (maternal allele dissomy and maternal imprinting) and the BC of Patient 2 presented *MEST* over-expression, suggesting the loss of imprinting. Similar mechanism has been described for *IGF2* (maternal imprinting) in colon cancer cells^43^.

Patient 1 and her son also presented a rare deletion of 9q22.31 encompassing the genes *CENPP* and *OGN*. The absence of OGN protein has been associated to colorectal carcinogenesis^44^, yet no alterations within these genes have been described as associated with cancer development risk. Three additional rare alterations were detected in Patient 2: two losses covering no genes on chromosomes 8q11.21, also present in her mother and one of her sons, and Xp25; as well as a cnLOH of chromosome 19p13.11-p13.2 encompassing 161 genes. Copy number aberrations on 19p13.2, including *PKN1, SMARCA4* and *LYL1* genes have been associated with triple negative BC and AM^45^,^46^.

Potential weakness of this study was the limited access of other relatives with cancer. In addition, neither histologically normal samples to evaluate the mosaicism nor other tumor samples from the probands were available. Nevertheless, the germline rearrangements detected in both probands, the cnLOH and deletion, are very large and encompass several TSGs previously associated with neoplasms, including BC, making this region an important candidate for MPCs risk.

In summary, we identified two large genomic rearrangements of chromosome 7q (mosaic loss and cnLOH) in two unrelated patients with MPCs, both presenting triple negative breast neoplasms and family history of cancer. The genomic analysis of the BC sample from Patient 2 indicated that the 7q region is prone to genomic instability and may include genes related to cancer growth and progression. The whole gene expression analysis of this BC revealed deregulated genes in 7q cnLOH region, including several cancer-related genes, giving additional support that genomic alterations of 7q have functional role in the tumor risk development.

## Methods

### Patients

The two probands (Patient 1 and Patient 2) included in this study were selected from a cohort of 27 MPC patients negative for pathogenic mutations in the *TP53, BRCA1* and *BRCA2* genes. Clinicopathological features of each individual were re-evaluated to exclude recurrence or metastases, confirming the diagnosis of multiple primary tumors. Sixteen of the 27 patients fulfilled criteria for hereditary cancer syndromes, including LFS (three cases), HBOC (five cases) and LFS/HBOC simultaneously (two cases)^27^,^47^. Female BC was the most common tumor type found in this set of MPC patients (*N* = 10) (Supplementary Table S5).

Additionally, five children (two of Patient 1 and three of Patient 2), the mother and the breast cancer tissue of Patient 2 were evaluated. This study was approved by the Human Research Ethics Committee of A.C. Camargo Cancer Center. Sao Paulo, Brazil (CEP No. 1726112). Written informed consent was obtained from all participants prior to sample collection. All experiments were carried out in accordance with the approved guidelines.

**Figure 2:**
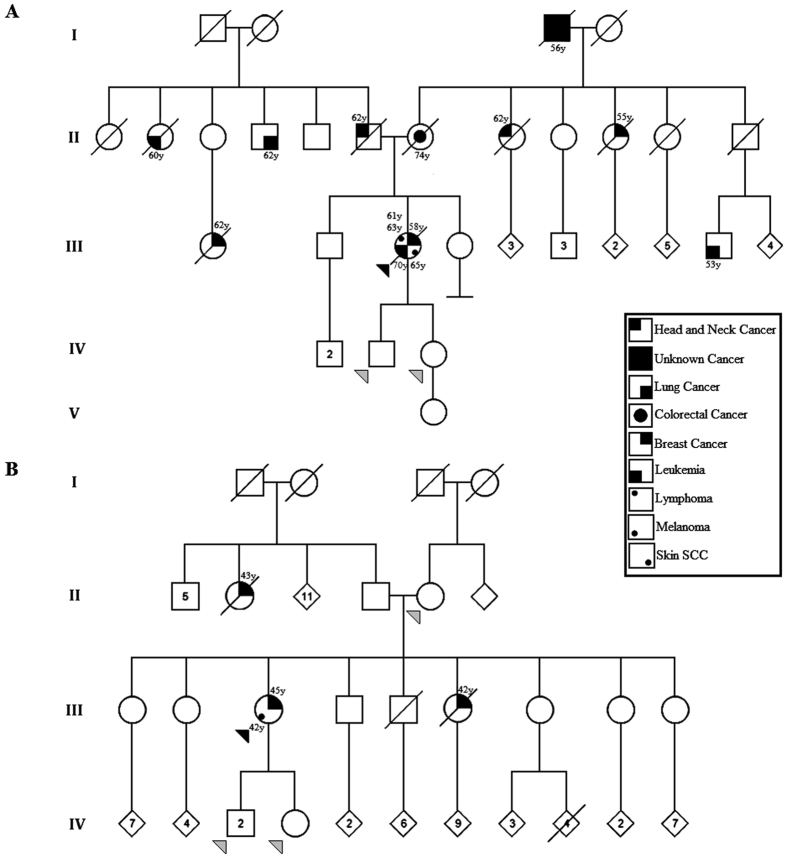
Pedigrees of Patient 1 (**A**) and Patient 2 (**B**). The patients fulfilled criteria for HBOC and were negative for germline mutations in the *BRCA1, BRCA2* and *TP53* genes. The black and grey arrows indicate the probands and the relatives submitted to molecular karyotyping analysis, respectively. The numbers above or below the symbols of individuals with cancer represent age at diagnosis. SCC: Squamous cell carcinoma.

Patient 1 was first diagnosed with triple-negative ductal carcinoma *in situ* on the right breast at 58 years of age. She underwent a segmental mastectomy, axillary lymphadenectomy and radiotherapy. At age 61, she presented with lymphocytic lymphoma, for which, she received chemotherapy and underwent axillary dissection. Orbital non-Hodgkin lymphoma of the left eye was diagnosed at 63 years of age, which was enucleated and treated with chemotherapy. At age 65, a squamous cell carcinoma of the lower lip was diagnosed and completely resected. Furthermore, the patient also developed chronic myeloid leukemia, dying at the age of 70. The patient met the criteria for HBOC (Fig. 2A)^27^. Multiple relatives from both the paternal and maternal sides of the family had been diagnosed with different neoplasms. Both of her children (one son and one daughter with 36 and 44 years age, respectively) have no cancer diagnosed in the last follow-up (November 2016).

Patient 2 developed *in situ* melanoma of the left foot at 42 years of age, and was treated by surgical resection. Three years later (aged 45) she was diagnosed with invasive ductal carcinoma of the left breast, characterized as pT2N1M0, triple-negative and with p53 positivity in 90% of the tumor cells. The patient was treated with modified radical mastectomy and adjuvant chemo-radiotherapy. The patient’s sister and paternal aunt both died from breast cancer at ages 42 and 43, respectively. The family also fulfilled the criteria for HBOC (Fig. 2B)^27^. To date, none of her three children (two sons and one daughter) have developed any neoplasms (aged 32, 33 and 37 years, respectively, November 2016).

### Molecular cytogenetic analysis

Genomic DNA was extracted from the peripheral blood of the probands, the mother of Patient 2, and five children of both probands following the standard protocol of the Gentra Puregene Blood Kit (Qiagen, Valencia, Ca, USA). Genomic DNA of the breast tumor tissue of Patient 2 was isolated using the phenol-chloroform method. Molecular karyotyping was carried out for all samples using the CytoScan HD Array (Affymetrix, Santa Clara, CA, USA) following the manufacturer’s instructions. The detection of CNVs and cnLOHs was performed using the Chromosome Analysis Suite (ChAS) software v.3.1 (Affymetrix). The criteria used for analysis considered at least 50 markers for gains/mosaic gains, 25 for losses/mosaic losses and cnLOHs with a minimum of 5 Mb. The alterations were visually confirmed, with poor quality data excluded. The CNVs detected were compared to the Affymetrix Database of Variants (aDGV), consisting of 2,421 phenotypically healthy individuals evaluated by Cytoscan HD, as well as to the Database of Genomic Variants (DGV, http://dgv.tcag.ca/dgv/app/home, updated in May 2016). CNVs with a frequency <0.5% in the aDGV and <0.05% in the DGV were considered rare. The microarray data are accessible at NCBI’s Gene Expression Omnibus (GEO, http://www.ncbi.nlm.nih.gov/geo/) database, accession number GSE 77138.

### Transcriptome analysis

Total RNA was isolated from the BC tissue of Patient 2 using the RNeasy Mini Kit (Qiagen, Valencia, Ca, USA), according to the manufacturer’s instructions. The Human Universal Reference RNA (Stratagene, Santa Clara, USA) was used as a reference sample for comparison with the tumor tisue. All samples were analyzed in duplicate. Whole genome gene expression analysis was assessed using the Human Transcriptome Array (HTA) 2.0 platform (Affymetrix) following the manufacturer’s recommendations. Microarray data were normalized with the Expression Console software (Affymetrix). Differentially expressed genes were obtained with the Transcriptome Analysis Console (TAC) software v3.1 (Affymetrix), considering the ANOVA test (*P* < 0.05) and fold change (FC) >1.5 or <−1.5. We limited our analysis to genes mapped to cnLOH of 7q detected in Patient 2. The gene expression data is also available in the GEO database (GSE 77138).

## Additional Information

**How to cite this article**: Villacis, R. A. R. *et al*. Germline large genomic alterations on 7q in patients with multiple primary cancers. *Sci. Rep.* **7**, 41677; doi: 10.1038/srep41677 (2017).

**Publisher's note:** Springer Nature remains neutral with regard to jurisdictional claims in published maps and institutional affiliations.

## Supplementary Material

Supplementary Figure S1

Supplementary Table S1

Supplementary Table S2

Supplementary Table S3

Supplementary Table S4

Supplementary Table S5
