# Supplementary figures and images for: Germline large genomic alterations on 7q in patients with multiple primary cancers
Source: Sci Rep. 2017 Jan 31;7:41677. doi: 10.1038/srep41677 (PMC5282589; doi:10.1038/srep41677)

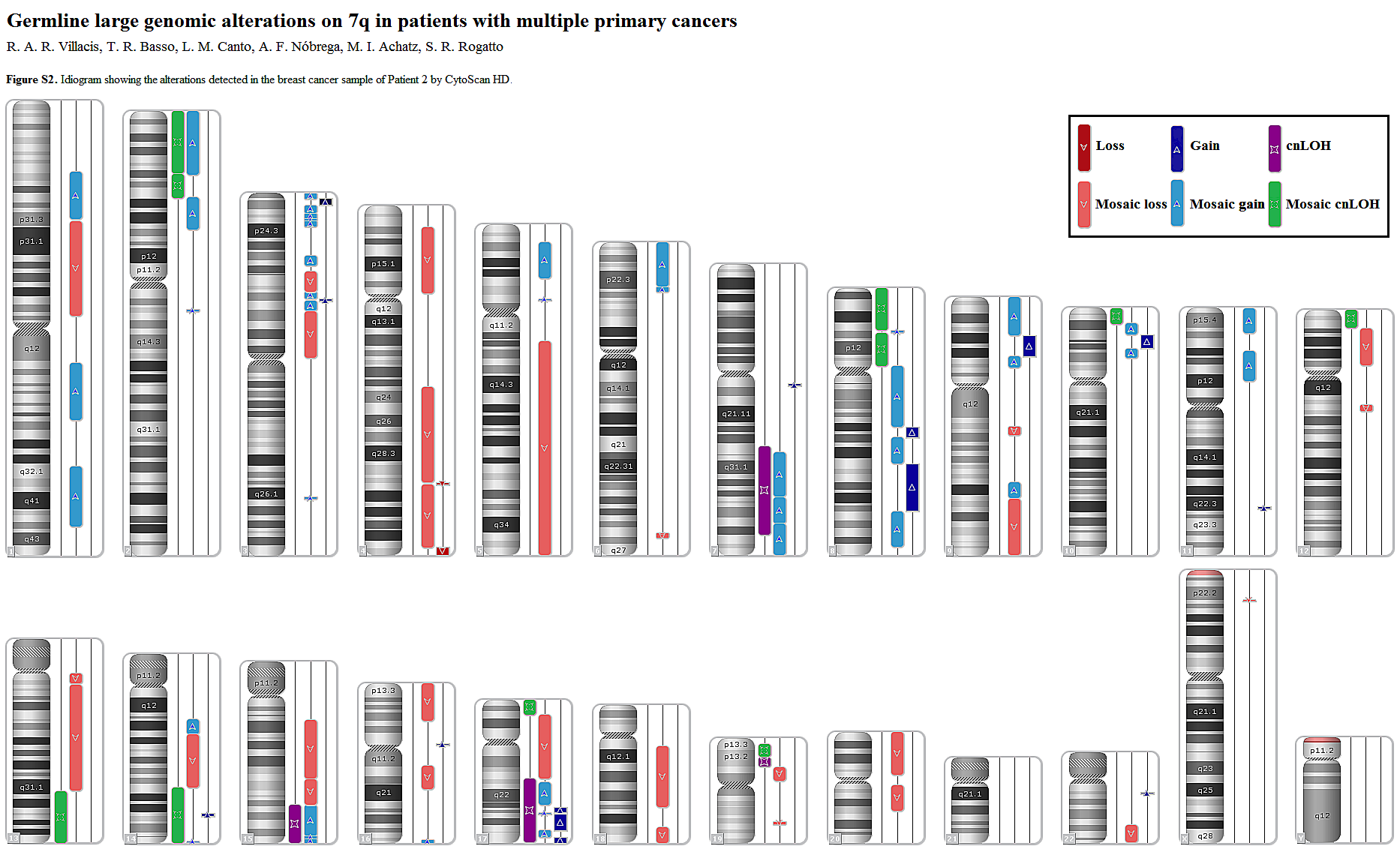

Supplement: Supplementary Figure S1 [file srep41677-s1.tiff]
